# Aspirin synergizes with mineral particle-coated macroporous scaffolds for bone regeneration through immunomodulation

**DOI:** 10.7150/thno.85946

**Published:** 2023-08-15

**Authors:** Ni Su, Cassandra Villicana, Carl Zhang, Jeehee Lee, Sauradeep Sinha, Andrew Yang, Fan Yang

**Affiliations:** 1 Department of Orthopaedic Surgery, Stanford University School of Medicine, Stanford, CA, 94305, USA.; 2 Department of Bioengineering, Stanford University School of Medicine, Stanford, CA, 94305, USA.

**Keywords:** mineral particles, osteogenesis, bone resorption

## Abstract

**Rationale:** Mineral particles have been widely used in bone tissue engineering scaffolds due to their osteoconductive and osteoinductive properties. Despite their benefits, mineral particles can induce undesirable inflammation and subsequent bone resorption. Aspirin (Asp) is an inexpensive and widely used anti-inflammatory drug. The goal of this study is to assess the synergistic effect of Asp and optimized mineral particle coating in macroporous scaffolds to accelerate endogenous bone regeneration and reduce bone resorption in a critical-sized bone defect model.

**Methods:** Four commonly used mineral particles with varying composition (hydroxyapatite v.s. tricalcium phosphate) and size (nano v.s. micro) were used. Mineral particles were coated onto gelatin microribbon (µRB) scaffolds. Macrophages (Mφ) were cultured on gelatin µRB scaffolds containing various particles, and Mφ polarization was assessed using PCR and ELISA. The effect of conditioned medium from Mφ on mesenchymal stem cell (MSC) osteogenesis was also evaluated* in vitro*. Scaffolds containing optimized mineral particles were then combined with varying dosages of Asp to assess the effect in inducing endogenous bone regeneration using a critical-sized cranial bone defect model. *In vivo* characterization and *in vitro* cell studies were performed to elucidate the effect of tuning Asp dosage on Mφ polarization, osteoclast (OC) activity, and MSC osteogenesis.

**Results:** Micro-sized tricalcium phosphate (mTCP) particles were identified as optimal in promoting M2 Mφ polarization and rescuing MSC-based bone formation in the presence of conditioned medium from Mφ. When implanted *in vivo*, incorporating Asp with mTCP-coated µRB scaffolds significantly accelerated endogenous bone formation in a dose-dependent manner. Impressively, mTCP-coated µRB scaffolds containing 20 µg Asp led to almost complete bone healing of a critical-sized cranial bone defect as early as week 2 with no subsequent bone resorption. Asp enhanced M2 Mφ polarization, decreased OC activity, and promoted MSC osteogenesis in a dosage-dependent manner *in vivo*. These results were further validated using *in vitro* cell studies.

**Conclusions:** Here, we demonstrate Asp and mineral particle-coated microribbon scaffold provides a promising therapy for repairing critical-sized cranial bone defects via immunomodulation. The leading formulation supports rapid endogenous bone regeneration without the need for exogenous cells or growth factors, making it attractive for translation. Our results also highlight the importance of optimizing mineral particles and Asp dosage to achieve robust bone healing while avoiding bone resorption by targeting Mφ and OCs.

## Introduction

Critical-sized bone defects cannot heal on their own, and tissue engineering offers a promising strategy to regenerate lost bone tissues 1. To enhance bone repair, calcium phosphate (CaP)-based minerals have been widely used due to their osteoconductive and osteoinductive functions 2-4. Different types of CaP with varying physical and biochemical cues have been exploited 5, 6 and combined with biodegradable scaffolds to fill defects and induce bone regeneration 7-9. Despite their strong osteoconductive properties, CaP minerals can also trigger undesirable inflammatory responses and cause subsequent bone resorption 10-12. Macrophages (Mφ) are one of the primary responders to CaP *in vivo*. CaP minerals can induce M1-Mφ polarization and release of excessive inflammatory cytokines 13-15. The CaP-induced inflammatory response can further inhibit stem cell osteogenesis 16 and activate osteoclasts (OCs) 17, 18, leading to bone loss due to imbalanced bone formation and resorption 19. To minimize the undesirable inflammatory responses of CaP minerals, it is critical to evaluate the immune response towards varying types of CaP and further exploit immunomodulatory strategies to enhance CaP-mediated bone regeneration.

Engineering biomaterials to induce immune responses that favor tissue regeneration has emerged as a promising strategy for regenerating various tissues, including muscle and bone 20, 21. To reduce CaP-induced inflammation, recent studies have explored optimizing biophysical properties 22, heparin coating 23, or co-delivery with exogenous mesenchymal stem cells (MSCs) 24. Delivery of immunomodulators represents another strategy to modulate immune cell response to treat Musculoskeletal injuries and diseases 25, 26. In particular, Asp is an attractive drug for immunomodulation, given its low cost and wide use as a nonsteroid anti-inflammatory drug in clinical settings. Asp can modulate multiple cell types in the bone niche, including Mφ, OCs, and MSCs. Asp has been shown to inhibit M1- Mφ polarization and OC activation by blocking the activation of nuclear factor-κB (NFκB) and receptor activator of nuclear factor kappa-B ligand (RANKL)-mediated signaling pathways 27, 28. Asp has also been shown to improve MSC osteogenesis through enhancing RUNX2 and Wnt-signaling 29, 30. Co-delivery of Asp and MSCs has been shown to enhance bone regeneration *in vivo* 31-33. Combining Asp with CaP in hydrogels has shown some promise in inducing endogenous bone repair, but the efficacy remains limited, with around 52% of bone defects filled after 12 weeks 34.

To overcome the above limitations, the goal of this study is to develop a macroporous scaffold containing Asp and optimized CaP particles to induce robust and rapid endogenous bone regeneration in a mouse critical-sized cranial defect model. We have chosen a gelatin-based microribbon (μRB) scaffold due to its macroporosity and advantages over nanoporous hydrogels in supporting bone formation *in vivo* 35. We hypothesized that coating μRB scaffolds with varying CaP particle composition and size would induce different Mφ-MSC crosstalk *in vitro*, and the optimized CaP particle would synergize with Asp to accelerate acellular μRB scaffold-mediated bone regeneration. Harnessing a Mφ conditioned medium treatment model* in vitro*, we identified micro-sized tricalcium phosphate particles (mTCP) as the leading CaP particle that has the least inflammatory response on Mφ while inducing robust MSC osteogenesis. When transplanted *in vivo*, Asp synergized with mTCP-μRB induced rapid and stable bone regeneration, achieving almost 100% filling of the critical-sized bone defects as early as week 2. Thorough characterization of *in vivo* tissue samples followed by *in vitro* cell studies further highlights the dosage-dependent effect of Asp on enhancing M2 Mφ polarization, decreasing OC activity, and enhancing MSC osteogenesis.

## Methods

### Chemicals and reagents

Lithium phenyl-2,4,6-trimethylbenzoylphosphinate (LAP), lipopolysaccharides (LPS), aspirin, Tartrate resistant acid phosphatase (TRAP) staining kit, and porcine gelatin were purchased from Sigma Aldrich. Tumor necrosis factor (TNF)-α and interleukin (IL)-10 DuoSet ELISA kits were purchased from R&D systems. Antibodies for immunostaining, including iNOS, CD206, osteocalcin (OCN), and CD31 were purchased from Invitrogen. All cytokines were purchased from PeproTech. Cell culture reagents, including Dulbecco's Modified Eagle Media (DMEM), minimum essential medium (MEM)-α, fetal bovine serum (FBS), penicillin-streptomycin (P/S), and DPBS were purchased from Gibco. For calcium phosphate particles, nano-size hydroxyapatite (HA) and nano-/micro-sized tricalcium phosphate particles (TCP) were purchased from Sigma Aldrich. Micro-sized HA particles were purchased from HiMed Inc.

### Cell culture

Mouse MSCs were isolated from the bone marrow of female Balb/c mice (JAX, USA). MEM-α was supplemented with 10% MSC-certified FBS and 1% P/S to culture MSCs. To prepare osteogenic media (OM), DMEM was supplemented with 10% FBS, 1% P/S, 100 nM dexamethasone (Sigma Aldrich), 10 mM beta glycerol phosphate disodium salt (Sigma Aldrich), and 50 μg/mL of ascorbic-2-phosphate (Sigma Aldrich). RAW 264.7 macrophage (ATCC) were cultured in DMEM supplemented with 10% FBS and 1% P/S.

### Fabrication of CaP-coated gelatin μRB scaffolds

Gelatin μRBs were fabricated by wet spinning, as previously reported 35. Gelatin was first dissolved in dimethyl sulfoxide (18% w/v), and the solution was ejected into a stirring alcohol bath to form a ribbon shape. Gelatin μRBs were conjugated with methacrylic acid N-hydroxysuccinimide ester overnight (Sigma Aldrich), followed by glutaraldehyde fixation. The μRBs were washed in water multiple times and lyophilized for -20°C storage until use. Sterile CaP solution was made at 5% (w/v) in PBS and autoclaved. CaP, PBS, and LAP were measured to reach a final concentration of 10% w/w of ribbons with 3.5% (w/v) CaP and 0.1% (w/v) LAP in the PBS portion. To create the scaffolds, a sandwich method was used in which hydrated μRBs were placed between two glass slides with a 0.5 mm spacer and clipped down to create the desired thickness. The mold was placed under UV light for 2.5 minutes on each side before punching out at desired diameter.

### Characterizing scaffold morphology and mechanical properties

To assess scaffold morphology, samples were characterized using scanning electron microscopy. Acellular scaffolds were briefly rinsed in distilled water after fabrication and then lyophilized. Dried scaffolds were fixed on the probe using colloidal silver liquid (Electron Microscopy Sciences). To provide a conductive surface layer, the scaffolds were coated with a thin layer of AuPd using a Cressington 108 Auto Sputter Coater for 120 s with a current of 20 mA. The samples were then imaged using a Magellan XHR SEM operated at 3 kV with a probe current of 50 pA. The scaffolds were then examined at 120× and 500× magnification.

To characterize the mechanical property of scaffolds, rheological testing was performed using a Discovery HR-2 hybrid rheometer (TA Instruments), as previously reported 36. Briefly, scaffolds of 0.5 mm thickness and 8 mm diameter were made and equilibrated in PBS overnight. Scaffolds were placed onto the rheometer base plate, and an 8mm top plate was immediately lowered onto the sample. A time sweep was performed at an amplitude of 1% shear strain and 1 rad s-1 frequency for 5 min at 37^o^C. Young's modulus (E) was calculated using the following equation:

E=2(1+ν) G*

Poisson's ratio (ν) is assumed to be 0.5, and G* is the complex modulus found using the equilibrium values of storage and loss moduli from the time sweep at 1% shear strain and 1 rad s^-1^ frequency. G* was calculated using the following equation:

G*=(G'^2^+ G''^2^)^1/2^

### Assessing Mφ polarization *in vitro*

RAW 246.7 Mφ were primed to M1 phenotype with 100 ng/mL LPS and 10 ng/mL interferon (IFN)-γ for 1 hour in tissue culture plates. Primed M1 Mφ were seeded on top of CaP-coated or non-CaP-coated μRB scaffolds. After 24 hours, Mφ seeded on varying CaP-μRB scaffolds were collected in Trizol for RNA extraction. Reverse transcription was performed using SuperScript™ III One-Step RT-PCR System (Invitrogen), and qPCR was performed using Power SYBR Green PCR Master Mix (Applied Biosystems). Primer sequences used for qPCR are listed in Table S1. Conditioned medium (CM) was collected from Mφ seeded on varying CaP-μRB scaffolds after 24-hour culture, centrifuged at 3000 g for 5 min, and filtered through 0.22 μm filter to remove the cell debris. ELISA was used to measure cytokine secretion from the Mφ CM collected in various conditions. For CM treatment studies, Mφ CM was stored at -80°C until use.

### Assessing MSC/Mφ crosstalk using conditioned medium treatment model

Mφ CM was collected from varying mineral coating conditions as described above. CM was mixed with OM at a 1:4 volume ratio and was applied to MSCs seeded on μRB scaffolds with the same mineral cues. For no CM controls, Mφ growth medium was used to mix with OM, instead of CM. The medium was refreshed twice a week, and scaffolds were fixed on day 16 and sectioned for alizarin red S (ARS) and Trichrome staining. Osteocalcin (OCN) immunostaining was performed at week 1 to determine bone matrix deposition. Specifically, tissue slices were treated with 0.3% Triton X-100 for 10 min at room temperature, followed by blocking with 10% bovine serum albumin (BSA) in PBS for 1 hour. Tissue slides were incubated with primary OCN antibody at 4°C overnight. Goat anti-rabbit secondary antibody (A11011, Invitrogen) and Hoechst 33342 were stained for 1 hour at room temperature. Images of tissue slices were taken with a Keyence microscope and quantified with ImageJ software. For the gene expression study, samples were harvested at week 1, and osteopontin (OPN) was evaluated by qPCR to determine the MSC osteogenesis.

### Fabricating Asp-loaded mTCP-μRB scaffolds for *in vivo*

Aspirin was incubated with mTCP particles in PBS solution for an hour on a rotator to allow Asp loading through physical interactions. Asp and mTCP were mixed at a mass ratio of 1:15 or 1:7.5 to achieve varying Asp dosage, equivalent to 10 or 20 μg Asp per scaffold/defect. Further increasing the amount of Asp incorporated in the scaffold would significantly compromise the crosslinking efficiency of scaffolds, leading to poor scaffold integrity (data not shown). As such, we chose 20 μg Asp/scaffold as the highest dosage of Asp for the *in vivo* study. The Asp-loaded mTCP was then added to gelatin μRBs and crosslinked into the scaffold under UV light as described above. To determine the Asp release kinetics, Asp-loaded mTCP-μRB scaffolds were incubated in PBS solution, and the released Asp was measured at different time points via UV-Vis spectrophotometer at 227 nm wavelength using NanoDrop (Thermo Fisher Scientific).

### Animal model and *in vivo* group design

Animal studies were approved by Stanford University's Administrative Panel on Laboratory Animal Care (APLAC 28583) and followed the Guidelines for the Care and Use of Laboratory Animals. Cranial defect surgery was performed on female Balb/c mice (JAX) at the age of week 6. Mice were anesthetized with 2.5% isoflurane during surgery, and a skin incision on the head was made to expose the skull. Periosteum was removed, and a trephine drill was used to create a circular defect with a diameter of 3.5 mm on the right side of the parietal bone. A 3.5 mm acellular μRB scaffold was used to fill the defect, and the skin incision was sutured. A total of three groups were included in the study 1) mTCP-μRB scaffolds, 2) 10 μg ASP loaded mTCP-μRB scaffolds, and 3) 20 μg ASP loaded mTCP-μRB scaffolds. Four replicates were included in each group.

### Monitoring bone regeneration using MicroCT

Bone regeneration of cranial defects in mice was assessed at week 1, 2, 3, and 6 using MicroCt TriFoil eXplore CT 120 (TriFoil Imaging, Chatsworth, CA). Scanning parameters were set at a high dose of 250 mGy, 135 HU noise. Before being placed in the scanner, mice were anesthetized with isoflurane. MicroCt images were acquired and reconstructed with eXplore Evolver and Reconstruction Interface software. A cylindrical region of interest (ROI) (X=3.5 mm, Y= 3.5 mm, Z=0.5 mm) was drawn onto the cranial defect. The bone volume and bone mineral density of the ROI were calculated with GEHC MicroView 2.2 (Parallax Innovations Inc, Ilderton, Canada).

### Histological analyses

Mice calvarias were harvested at week 2 for mechanistic studies or at week 6 for bone regeneration study. The samples were fixed in 4% paraformaldehyde (PFA) for 3 days and demineralized in 0.5M ethylenediaminetetraacetic acid (EDTA) for 3 weeks. Samples were then placed in 30% sucrose PBS solution overnight, and later embedded in OCT for future cryosectioning. Sections were taken at a thickness of 12 μm. For samples harvested at week 6, H&E staining was used to visualize tissue morphology. Trichrome staining was used to visualize collagen deposition. OCN IF staining was performed to characterize bone matrix formation and CD31 IF staining was performed to evaluate endothelial cell vascularization at week 6. For samples harvested at week 2, TRAP staining was used to evaluate bone remodeling according to manufacturer's instructions. OCN IF staining was performed to characterize bone matrix formation. IF staining of F4/80, iNOS, and CD206 was performed to determine the total Mφ number and their phenotype at week 2. For IF image analysis, 8 tissue sections per biological replicate for each group were imaged using a Keyence microscope and quantified with ImageJ software. For TRAP staining images, the purple color was extracted, and the area was quantified using ImageJ software.

### Validating the dosage effect of Asp using *in vitro* cell culture

To validate the effect of Asp dosages on various cell types* in vivo*, we cultured each cell type on mTCP-μRB scaffolds* in vitro* and treated with Asp with varying dosages (0, 200, 500, 750, and 1000 μg/mL). This *in vitro* dosage in the medium is equivalent to the *in vivo* dosage of 0, 4, 10, 15, and 20 μg Asp per scaffold/defect. For Mφ, RAW 264.7 Mφ were seeded on mTCP-μRB scaffolds and cultured in DMEM growth medium containing varying dosages of Asp. Mφ were primed with 100 ng/mL LPS for 6 hours to induce M1 polarization, and samples were collected after 24 hours of Asp treatment. Gene expression of M1/M2 Mφ markers and cytokine secretion was assessed as mentioned above.

To induce OC differentiation, RAW 264.7 Mφ was used, and the media was supplemented with 50 ng/mL RANKL and 20 ng/mL colony-stimulating factor (M-CSF). Cells were seeded on mTCP coated μRB scaffolds and treated with varying dosages of Asp. The medium was refreshed once on day 2, and samples were collected on day 4. Samples were sectioned for TRAP staining, and TRAP+ OC number was quantified using Image J. Samples were also treated with Trizol, and OC differentiation was evaluated using PCR for markers including TRAP, osteoclast-associated receptor (OSCAR), and cathepsin K (CTSK).

To assess the dosage effect of Asp on MSCs, cells were seeded on mTCP coated μRB scaffolds and cultured in OM supplemented with varying dosages of Asp for one week before analyses. OCN IF staining was performed to evaluate MSC bone formation. Osteogenic gene expression of alkaline phosphatase (ALP), bone morphogenetic proteins (BMP), and OCN were evaluated by qPCR.

### Statistical analyses

GraphPad Prism 9 was used to conduct statistical analysis. Statistical comparisons were performed by one-way ANOVA followed by Tukey test to compare selected data pairs. The values of p < 0.05 were considered statistically significant. Data values were shown as mean ± SD.

## Results and Discussion

### Morphology and mechanical properties of CaP-μRB scaffolds

**Figure 1:**
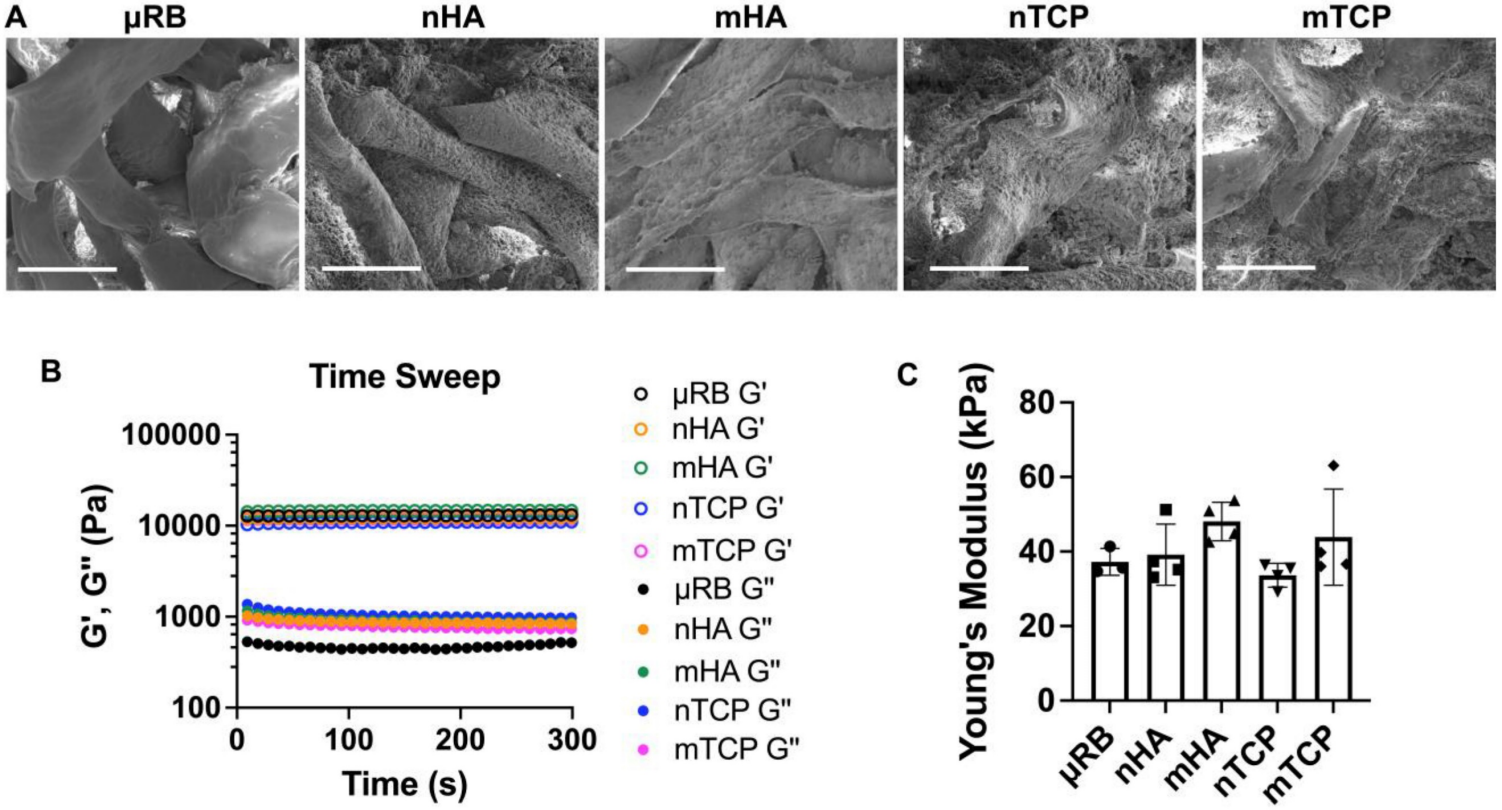
** Morphology and mechanical properties of CaP-μRB scaffolds.** (A) SEM imaging of μRB scaffolds coated with varying CaP particle types. (B) Storage modulus (G') and loss modulus (G'') of CaP-μRB scaffolds. (C) Young's modulus of CaP-μRB scaffolds. Scale bar: 100 μm. Data are represented as mean ± S.D. (n = 3~4/group).

Four types of CaP particles were evaluated, including two particle sizes (nano/micro) and two compositions of hydroxyapatite (HA) and tricalcium phosphate (TCP). All particles were coated onto μRB scaffolds via surface adsorption. First, we conducted SEM imaging to characterize the morphology and particle distribution of the mineral-coated μRB scaffolds. SEM confirms the macroporosity of the μRB scaffolds (Figure 1A) and shows a relatively uniform distribution of mineral particle coating. Nano-sized mineral particles have higher coverage of the μRB surface compared to micro-sized mineral particle coating. This may be caused by nano-sized mineral particles having a higher number of particles than micro-sized mineral particles, as the total mass of minerals was kept consistent among groups. We used rheology testing to measure the mechanical property of the scaffolds and to assess if mineral particle coating would affect the mechanical property of the resulting scaffolds. Our results demonstrate a plateaued storage modulus (G') and loss modulus (G''), in which the G'/G'' > 10, indicating the formation of solid-like hydrogels across all formulations (Figure 1B). All formulations exhibited comparable Young's Modulus to that of µRB scaffolds (37.23 ± 3.61 kPa), indicating that adding mineral particles did not significantly impact the mechanical property of the µRB scaffolds (Figure 1C).

### Varying CaP particles modulates Mφ polarization

**Figure 2:**
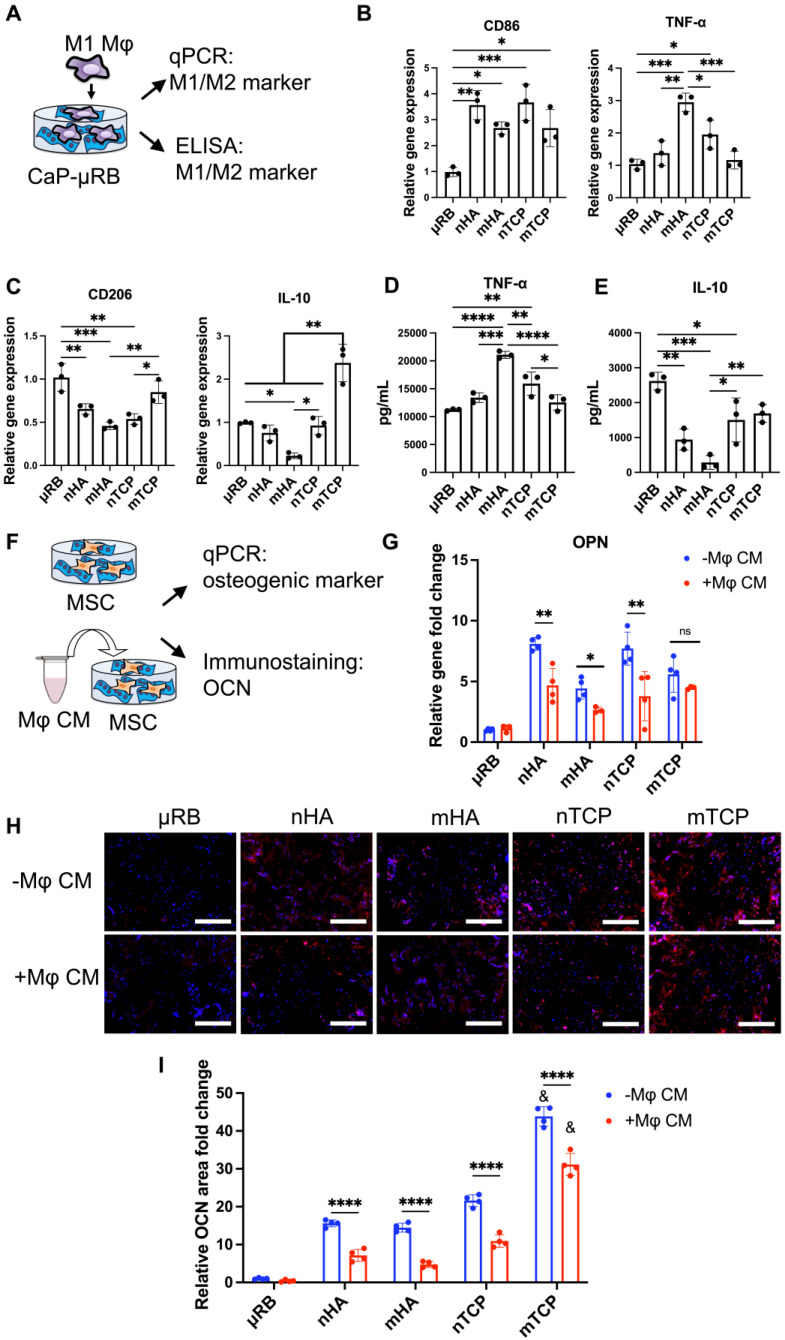
** Varying the composition (HA vs. TCP) and size of CaP particles (micro vs. nano) modulates Mφ polarization, and the corresponding effect of Mφ conditioned medium on MSC osteogenesis.** Tricalcium phosphate microparticle (mTCP) was identified as the lead formulation, which promotes M2 Mφ polarization and enhances MSC-based bone formation in the presence of conditioned medium from Mφ. (A) Schematics of experimental design. (B-C) Normalized gene expression of Mφ M1 (B) and M2 markers (C); (D-E) Quantification of TNF-α (D) and IL-10 (E) secreted by Mφ at 18 hr using ELISA. (F) Schematic of conditioned medium (CM) treatment on MSC osteogenesis and bone formation. (G) MSC osteogenic gene expression was evaluated at day 7 after encapsulation in CaP-μRB scaffolds with or without treatment of conditioned medium (CM) from Mφ cultured in the same CaP-μRB formulation. (H, I) Representative OCN staining (H), and OCN area quantification with or without Mφ CM treatment (I). Scale bar: 100 μm. Data are represented as mean ± S.D. (n = 3~4/group). *P < 0.05, **P < 0.01, ***P < 0.001, ****P < 0.0001. ns indicates not significant. & indicates comparison of mTCP with other groups, &P < 0.0001.

We then assessed the effects of varying CaP particle composition and size on Mφ polarization (Figure 2A). To mimic the acute inflammation after injury, Mφ were primed to M1 phenotype with IFN-γ and LPS and then seeded on top of CaP-μRB scaffolds to mimic Mφ infiltration *in vivo*. Compared to μRB with no CaP coating, all four CaP particles increased M1 marker gene expression (Figure 2B) while decreasing the gene expression of M2 markers (Figure 2C). Consistent with the trend in gene expression, ELISA showed CaP particles increased secretion of TNF-α and decreased IL-10 (Figure 2D-E). These results verify the known pro-inflammatory effect of CaP particles. Among four types of tested CaP particles, mHA is the most pro-inflammatory, as shown by the upregulation of TNF-α at gene expression and protein levels (Figure 2B, 2D). In contrast, mTCP is the least inflammatory, with low TNF-α secretion (Figure 2D) and increased expression and secretion of IL-10 (Figure 2C, 2E). As such, mTCP was identified to have the optimal immunomodulatory effects on Mφ polarization among the four types of CaP particles tested. These results demonstrate that varying the composition and size of CaP particles is an effective strategy to modulate the Mφ immune response. In our study, different mineral particles led to differential Mφ polarization. This may be due to several reasons. First, different CaP particles have different chemical compositions and sizes, which can directly change Mφ response. Furthermore, SEM images showed that different particle coating led to varying topographical cues of the μRB surface (Figure 1A). Previous studies have reported that varying the surface topography of mineral-coating can impact Mφ response 37. Last, TCP has a faster degradation rate than HA, which can also impact the immunomodulatory response of Mφ. Recent studies have examined the immunomodulatory effect of CaP. However, the results of these studies are hard to compare due to differences in other cultural conditions, and results can be contradictory. HA coating on tissue culture plates and HA ceramics was reported to polarize macrophages towards a pro-inflammatory phenotype 38, 39, whereas HA coating on porous titanium scaffolds showed an anti-inflammatory effect 40. These results suggest that the immunomodulatory effects of CaP are highly dependent on the form and topographical cues and must be investigated in a context-dependent manner.

### Varying CaP particles modulates MSC osteogenesis and MSC/ Mφ crosstalk

Next, we assessed how varying CaP particles directly affect MSC-based bone formation, as well as how CaP-induced Mφ phenotypic changes indirectly affect MSC bone formation through cytokine secretion. We adopted a conditioned medium (CM) treatment model, where CM from Mφ cultured in different CaP coating was mixed with osteogenic medium (20% CM and 80% OM) and used to treat MSCs seeded on μRB scaffold with the same CaP coating (Figure 2F). Compared to μRB alone, all CaP-coated groups demonstrated significant upregulation of osteopontin (OPN) and enhanced osteocalcin (OCN) deposition (Figure 2G-I), indicating the osteogenic function of CaP particles. Among the four types of CaP particles, mTCP showed the most robust OCN deposition (Figure 2H-I), while no differences were found in OPN gene expression (Figure 2G). When treated with Mφ CM, a significant decrease in OPN gene expression was found in all groups except for mTCP (Figure 2G). Mφ CM treatment led to a significant decrease in OCN in all CaP groups, compared to no CM treatment (Figure 2H-I). Mφ CM from mTCP coating induced a 31% decrease in OCN deposition, which is the least compared to other minerals, with a 54%, 67%, and 48% OCN decrease for nHA, mHA, and nTCP, respectively. A similar trend was confirmed by ARS staining, which showed Mφ CM from mTCP coating induced the least inhibition of MSC mineralization, while Mφ CM from mHA completely abolished MSC mineralization (Figure S1A). Collagen deposition was comparable among different CaP coatings and was not impacted by Mφ CM treatment significantly (Figure S1B). These results validated our hypothesis that varying CaP particles affect Mφ polarization differently, further impacting MSC bone formation via Mφ-MSC crosstalk. Based on the results of the Mφ polarization and CM treatment model, mTCP was selected as the optimal CaP particle, given that it had the least inflammatory response and robust osteogenic function.

### Asp synergizes with mTCP- μRB scaffold to accelerate bone regeneration *in vivo* in a dose-dependent manner

**Figure 3:**
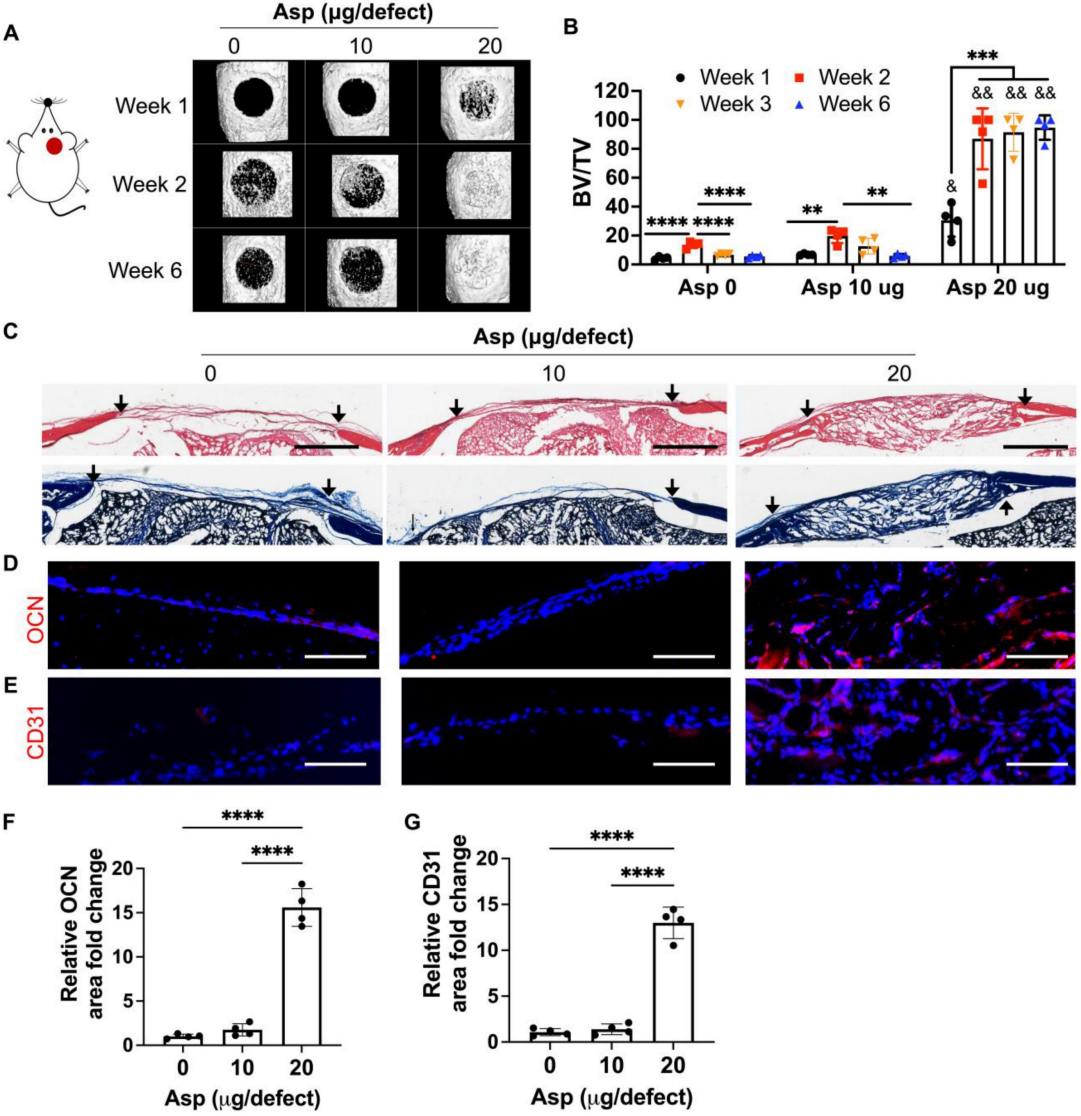
** Incorporating aspirin in mTCP-μRB scaffolds significantly accelerated endogenous bone formation in a dose-dependent manner *in vivo*. Lead formulation (20 µg/defect) led to rapid bone regeneration that completely filled the critical-sized cranial bone defect as early as week 2.** (A-B) Representative μCT images (A) and percentage of newly formed bone volume (B) in mouse cranial defects. BV: bone volume. TV: total volume. (C) Representative H&E and trichrome histology image of bone defects at week 6. Black arrow indicates the edges of defects. Scale bar: 1 mm. (D-E) Representative immunofluorescent staining images and quantifications of OCN+ area (D, F) and CD31+ endothelial cells (E, G) in the cranial defect at week 6. Scale bar: 100 μm. (n = 4/group). * indicates comparison within the same Asp dosage at varying time points. **P < 0.01, ***P < 0.001, ****P < 0.0001. & indicates comparison of 20 μg Asp with 0 μg and 10 μg Asp at each time point. &P < 0.01, &&P < 0.0001.

Now that we have identified mTCP as the optimal CaP particle, we next assessed the efficacy of co-delivery of Asp with mTCP-coated μRB scaffolds in inducing endogenous bone regeneration in a mouse critical-sized cranial defect model. Two dosages of Asp (10 μg and 20 μg per defect) were loaded to mTCP particles through physical adsorption, which was further incorporated into macroporous μRB scaffolds. Bright-field microscopic images showed that loading Asp to mTCP did not change the size and morphology of mTCP particles (Figure S2A). SEM imaging further confirms that the loading of Asp doesn't affect the macroporosity (Figure S2B) and the mechanical properties (Figure S2C-D) of the μRB scaffolds, compared to 0 Asp scaffolds. The* in vitro* release kinetics verified a gradual release of Asp from the scaffolds, with 65% and 81% of accumulated Asp release from 10 μg Asp/scaffold and 20 μg/scaffold by 12 days, respectively (Figure S3). MicroCT results showed that mTCP alone induced 21% new bone formation* in vivo* at week 2. However, this was followed by significant bone loss at week 3 and week 6, indicating excessive bone resorption (Figure 3A-B). Asp delivered at a lower dosage (10 μg/defect) did not show significant benefits, with a similar level of new bone formation as the 0 Asp control group (Figure 3A-B). Impressively, increasing Asp dosage to 20 μg/defect led to rapid endogenous bone regeneration, with bone defects filled up to 94% as early as week 2. The volume and bone mineral density of the newly formed bone in the 20 μg Asp group is significantly higher than 0 μg and 10 μg Asp groups at all time points assessed (Figure 3B and S4). Importantly, the 20 μg Asp group maintained stable bone volume over week 6 with no sign of bone resorption, leading to a 15-fold increase compared to the 0 μg and 10 μg Asp group at week 6. This indicates balanced osteogenesis and osteoclastogenesis in the defect niche in 20 μg Asp formulation. These results validated our hypothesis that Asp and CaP synergize to enhance bone regeneration and avoid excessive bone resorption. One previous study that harnesses Asp and TCP for treating bone defects achieved a less robust regeneration outcome compared to our study, with 52% new bone formation at week 12 *in vivo* 34. This highlights the importance of choosing optimal CaP formulation and Asp dosage to achieve maximal benefit. Consistent with our findings, previous studies that co-deliver MSC with Asp also showed that a critical Asp dosage needs to be met to achieve significant improvements in bone regeneration *in vivo* 30, 32.

To further assess the morphology and vascularization of newly formed bone, histology and immunostaining were performed. For material degradation, *in vitro* degradation assays could not predict *in vivo* degradation due to the lack of niche cues* in vivo*. To more accurately characterize the scaffold degradation *in vivo*, we used histology from harvested *in vivo* tissues to assess the degradation among groups. By week 2, µRB scaffolds were mostly intact in the cranial defects for all the groups (Figure S5). By week 6, the scaffolds were completely degraded in the 0 μg and 10 μg Asp groups and were replaced by a thin layer of fibrotic tissue (Figure 3C). In contrast, the macroporous µRB structures were still noticeable in the 20 μg Asp group, which is also filled with newly deposited tissues (Figure 3C). These results indicate 0 μg Asp and 10 μg Asp groups are subject to faster degradation *in vivo*, compared to a more balanced degradation and new tissue deposition in the 20 μg Asp group. The 20 μg Asp group also induced bone maturation and vascularization, as shown by the OCN and CD31 immunostaining (Figure 3D-G).

### Asp dosage effects on Mφ polarization

To further elucidate how Asp synergizes with mTCP-μRB to enhance bone regeneration, we characterized the effect of tuning Asp dosage on Mφ, OCs, and MSCs *in vivo*, and further validated the dosage effect of Asp using *in vitro* cell studies. We chose week 2 for *in vivo* tissue analysis, as it allows us to assess the early immune response and MSC activity before the dramatic bone loss after week 2, as shown by microCT (Figure 3B). For *in vitro* cell studies, Asp was supplemented in the media across a broad range of concentrations, with the highest concentration being 1000 μg/mL. The Asp concentration for *in vitro* studies was chosen to match the optimal dosage of Asprin identified from *in vivo* study (20 μg Asp per defect). Each scaffold in the defect contains 20 μg Asp within a volume of 4.8 μL, with around 25% Asp being released at day 1 as estimated by *in vitro* release kinetics (Figure S3). This converts to about 1042 μg/mL in Asp concentration, comparable to the highest Asp concentration for *in vitro* studies.

**Figure 4:**
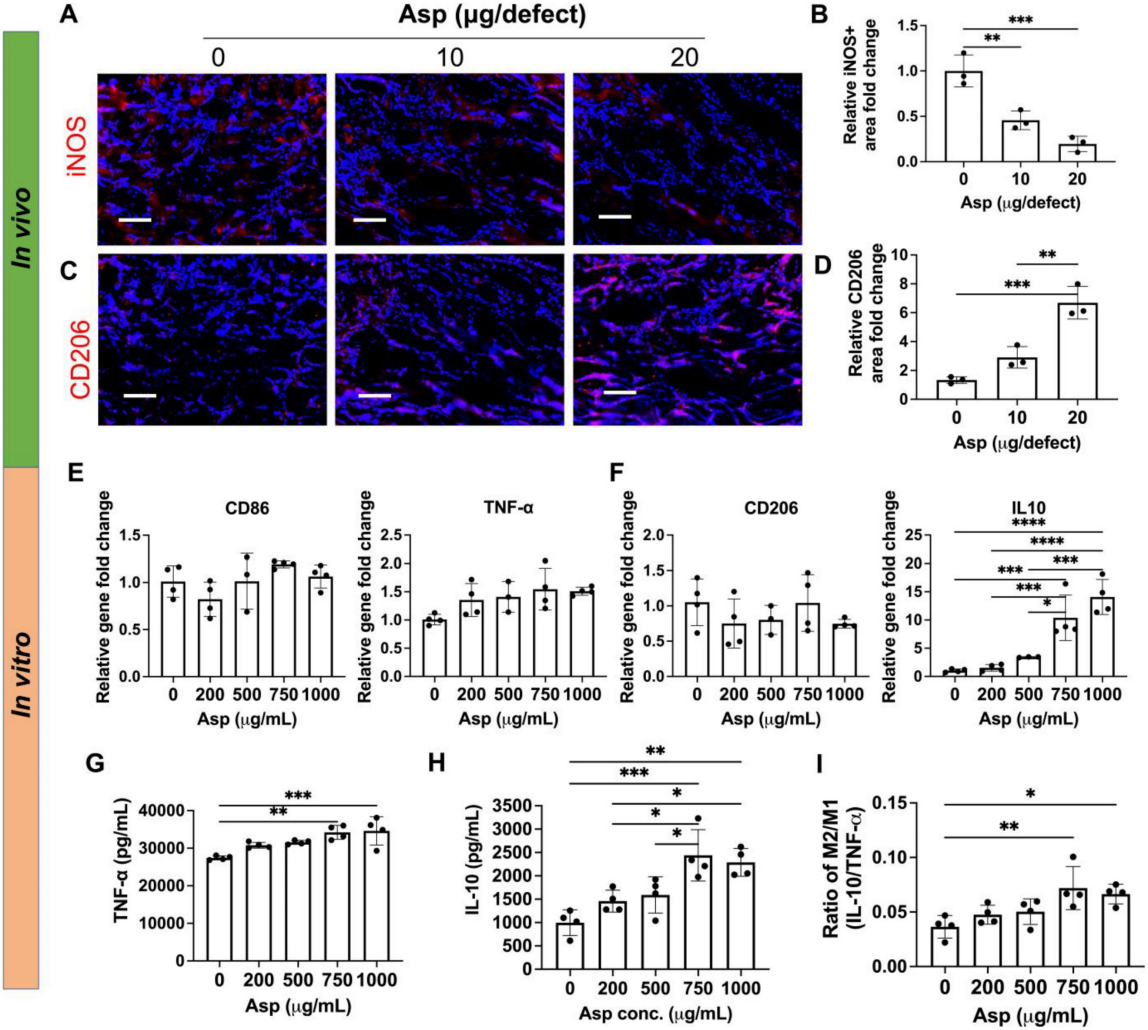
** Aspirin promoted M2 Mφ polarization *in vivo* in a dosage-dependent manner, which was validated using an *in vitro* model.** (A-D) Representative immunostaining images and quantification of iNOS+ M1 Mφ (A, B) and CD206+ M2 Mφ (C, D) in the cranial defect treated with 0/10/20 μg Asp-loaded mTCP-μRB scaffolds at week 2. Scale bar: 100 μm. (n = 3/group) (E-F) Normalized gene expression of M1 markers (E) and M2 markers (F) of Mφ; (G-I) ELISA measurement of TNF-α (G) and IL-10 (H) and IL-10/TNF-α ratio (I) secreted by Mφ seeded on the mTCP-μRB scaffolds treated with varying concentration of Asp *in vitro* after 24 hours. (n = 4/group). *P < 0.05, **P < 0.01, ***P < 0.001, ****P < 0.0001.

The effects of Asp on Mφ infiltration and phenotype were first evaluated. Adding Asp did not change the total number of infiltrated Mφ *in vivo*, as shown by the immunostaining of F4/80, a pan marker for Mφ (Figure S6). Asp led to decreased M1 (iNOS) and increased M2 (CD206) marker expressions at week 2 (Figure 4A-D). Importantly, increasing Asp dosage from 10 μg/defect to 20 μg/defect further decreased iNOS and increased CD206 (Figure 4A-D), indicating Asp supports Mφ transition from M1 to M2 in a dosage-dependent manner. Timely Mφ phenotypic transition from M1 to M2 has been recognized as a critical process for normal bone healing 41. As such, we speculate that Asp enhanced bone regeneration partially by facilitating the M1-to-M2 transition, which positively affects vascularization, new bone deposition, and remodeling. *In vitro* cell studies further validate the dosage-dependent effects of Asp on inhibiting M1 Mφ polarization. Increasing Asp dosage led to a significant upregulation of IL-10 gene expression (Figure 4F), while no significant change was observed in CD86, TNF- α, and CD206 (Figure 4E-F). ELISA showed increased release of both TNF-α and IL-10 for the top two Asp concentration groups (Figure 4G-H). Notably, the relative increase of IL-10 is greater than TNF-α, as shown by the increase in the ratio of IL-10/TNF-α (Figure 4I). These results indicate that high Asp dosages promote M2 Mφ polarization. It was observed that *in vivo* tissue analysis shows a stronger inhibition of M1 Mφ compared to *in vitro* cell studies, likely due to the crosstalk with other cell players in the bone niche. In general, both* in vivo* tissue characterization and *in vitro* cell studies indicate the dosage-dependent effects of Asp in facilitating Mφ polarization towards the pro-regenerative M2 Mφ phenotype.

A few recent studies have designed drug-loaded biomaterial scaffolds for bone regeneration via targeting immunomodulation. Some use growth factors (IL-4 and BMP-2) or glycopeptide 42, 43, which is costly, and glycopeptide has not been approved by FDA. Compared to the previous approaches, our design is more advantageous because Asp is an FDA-approved drug much cheaper than growth factors, thereby providing more advantages for clinical translation. Other studies have explored adding piezoelectric periosteum or gold nanoparticle (AuNP)-loaded silica nanoparticles to the scaffolds for bone regeneration 44, 45. However, the speed of bone regeneration *in vivo* is much slower, with only 40%~70% of bone defects filled by week 8. In contrast, our strategy led to rapid endogenous bone healing, with the critical bone defect almost completely filled as early as week 2. Together, these results demonstrate the novelty and advantages of our scaffold design compared to previously reported scaffolds.

### Asp dosage effects on Osteoclasts

**Figure 5:**
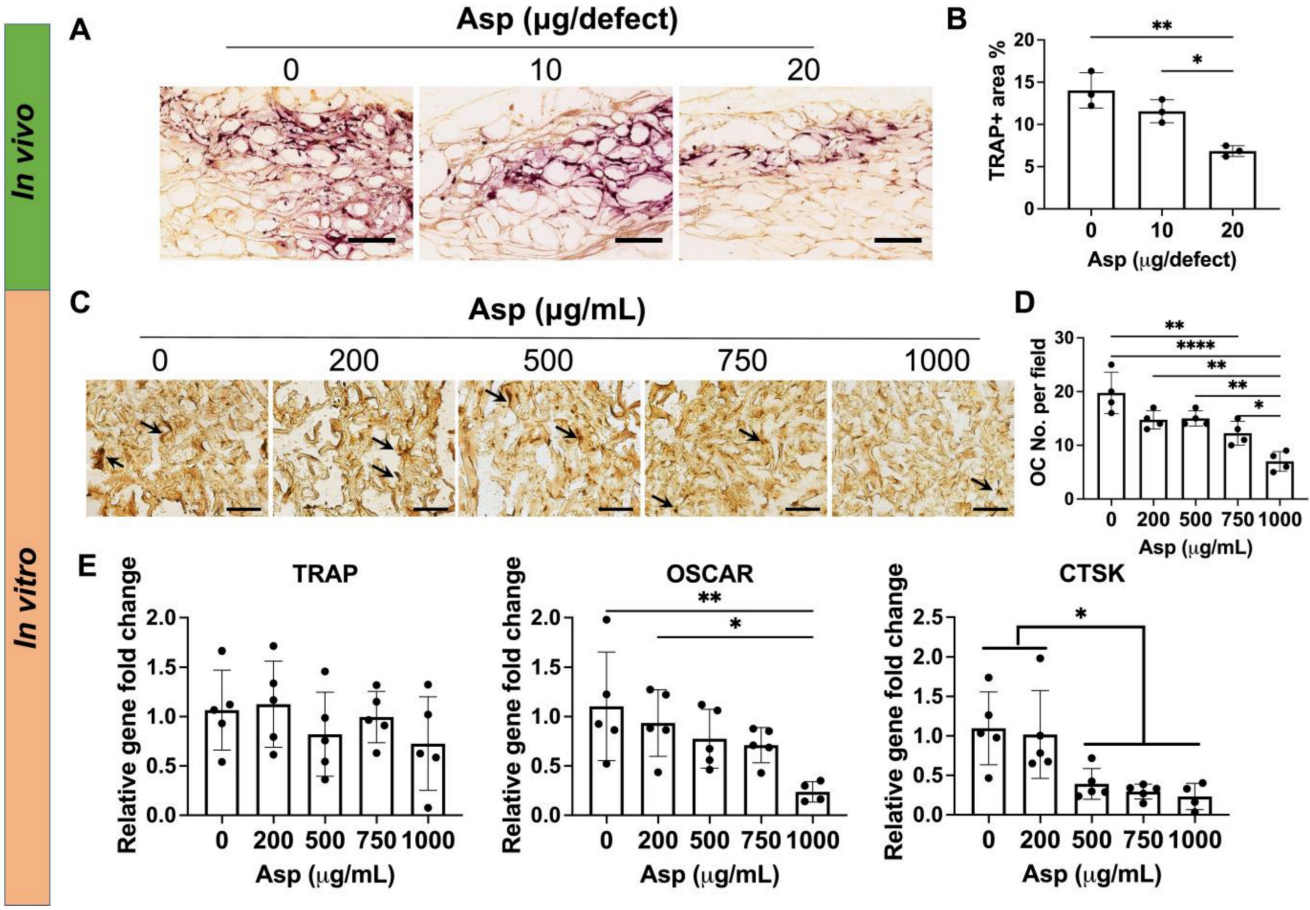
** Incorporating aspirin in mTCP-μRB scaffolds inhibited osteoclast activity *in vivo* and *in vitro* in a dosage-dependent manner.** (A-B) Representative TRAP staining images (A) and TRAP+ area quantification (B) in the cranial defect treated with 0/10/20 μg Asp-loaded mTCP-μRB scaffolds at week 2. (n = 3/group) (C-D) Representative TRAP staining images (C) and quantification (D) of OCs on the mTCP-μRB scaffolds treated with varying concentrations of Asp *in vitro* at day 5. (E) Normalized gene expression of OC markers *in vitro* at day 5. (n = 4/group). Scale bar in (A, C): 200 μm. *P < 0.05, **P < 0.01, ****P < 0.0001.

Given 20 µg Asp group avoided CaP particle-induced bone loss *in vivo* (Figure 3A-B), we hypothesized that Asp reduces OC activation and subsequent bone remodeling in a dose-dependent manner. As such, we then assessed the effects of Asp dosage on OC activity *in vivo* and *in vitro*. *In vivo* tissue analysis showed 20 μg Asp group significantly decreased TRAP+ OCs at week 2, compared to 0 μg and 10 μg Asp groups (Figure 5A-B). This may explain why the bone volume loss was observed in 0 μg and 10 μg Asp after week 2 but not in the 20 μg Asp group (Figure 3A-B). Previous studies showed that the presence of OC is indispensable for CaP-mediated bone regeneration, as depletion of OCs impeded CaP-induced bone formation 46. OCs can facilitate the release of ions from CaP and support osteoconductive function. However, CaP may also trigger excessive OC activation and bone resorption 19, As such, it is important to modulate OC activity to balance the speed of bone formation and bone resorption in CaP-induced bone regeneration, and the dosage and release kinetics of Asp is a critical factor to achieve desirable immunomodulation.

Asp-induced decrease in OC numbers* in vivo* may be contributed to two factors: decreased OC migration and decreased OC differentiation. We then harnessed the* in vitro* system to further decouple how varying Asp dosages directly affect OC differentiation. Mφ were seeded on mTCP-μRB scaffolds to mimic the inflammatory environment induced by mTCP and were treated with M-CSF and RANKL to induce OC differentiation for five days. Asp at varying concentrations was added to the media to assess the effects of Asp dosage on OC differentiation. TRAP staining and gene expression of OC differentiation were evaluated. TRAP staining shows that increasing Asp dosage decreased OC numbers in the mTCP-μRB scaffolds (Figure 5C-D). A similar trend was observed in the gene expression of OC differentiation markers, as OSCAR and CTSK expression levels decreased with higher Asp dosages (Figure 5E). Gene expression of TRAP showed no significant differences when varying Asp dosages (Figure 5E). The inconsistency between gene expression and TRAP staining could be explained by the fluctuation of gene expression during OC differentiation. In general, the trend of *in vitro* study correlates with *in vivo* study, supporting that high Asp dosage inhibits OC differentiation.

### Asp dosage effects on MSC osteogenesis

**Figure 6:**
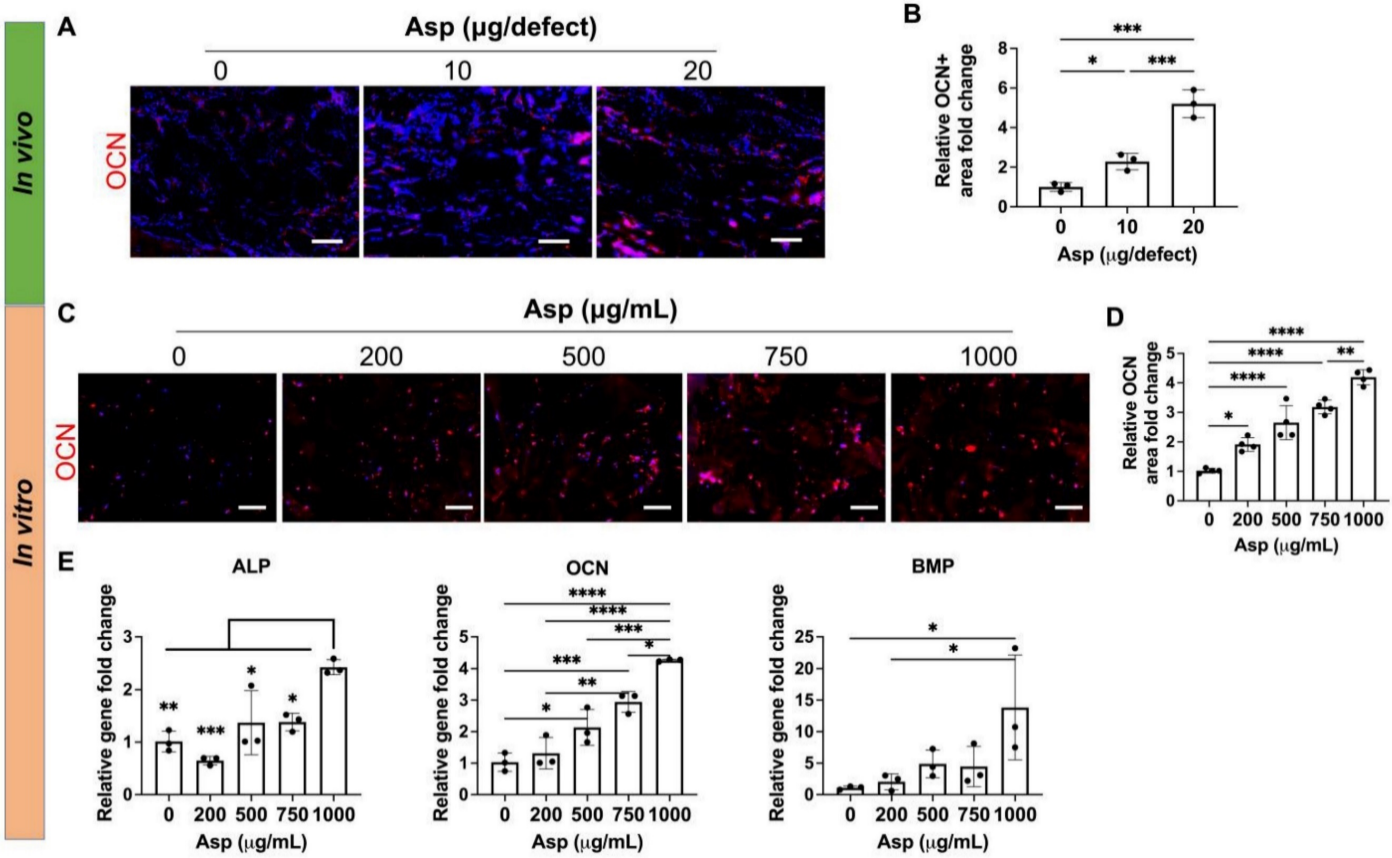
** Incorporating aspirin in mTCP-μRB scaffolds enhanced MSC osteogenesis *in vivo* and *in vitro* in a dosage-dependent manner.** (A-B) Representative immunostaining images (A) and quantification (B) of OCN+ MSC in the cranial defect treated with 0/10/20 μg Asp-loaded mTCP-μRB scaffolds at week 2. (n = 3/group) (C-D) Representative immunostaining images of OCN+ area (C) and quantification (D) on the mTCP-μRB scaffolds treated with varying concentrations of Asp *in vitro* at day 7. (E) Normalized gene expression of MSC osteogenic markers *in vitro* at day 7. (n = 4/group). Scale bar in (A, C): 100 μm. *P < 0.05, **P < 0.01, ***P < 0.001, ****P < 0.0001.

Given that Asp has been previously shown to enhance MSC osteogenesis on 2D tissue culture plates 30 and 3D scaffolds 47, we then assessed the direct effect of Asp on MSC osteogenesis in our 3D mTCP-μRB scaffold. Immunostaining of mature bone marker OCN showed Asp increased bone formation as early as week 2 *in vivo*, with the 20 μg Asp group showing the most robust bone formation (Figure 6A-B). Considering our approach is an acellular scaffold, it is impressive to observe robust MSC infiltration and bone matrix deposition as early as week 2, highlighting the synergistic efficacy of Asp plus mTCP-μRB formulation.* In vitro* MSC culture with increasing dosage of Asp showed a similar trend of enhanced OCN deposition (Figure 6C-D). Gene expression of osteogenic markers was also upregulated with increasing Asp dosage (Figure 6E). OCN gene expression is more sensitive to Asp dosage, showing a significant increase starting at 500 μg/mL, consistent with protein level assessment. In comparison, only the highest Asp dosage induced a significant increase in ALP and BMP gene expression. Taken together, our results validate that Asp promotes MSC osteogenesis in a dosage-dependent manner. The net increase of bone volume observed in the 20 μg Asp group over time is a combined result of both enhanced MSC osteogenesis and decreased bone remodeling mediated by OCs.

## Conclusions

**Figure 7:**
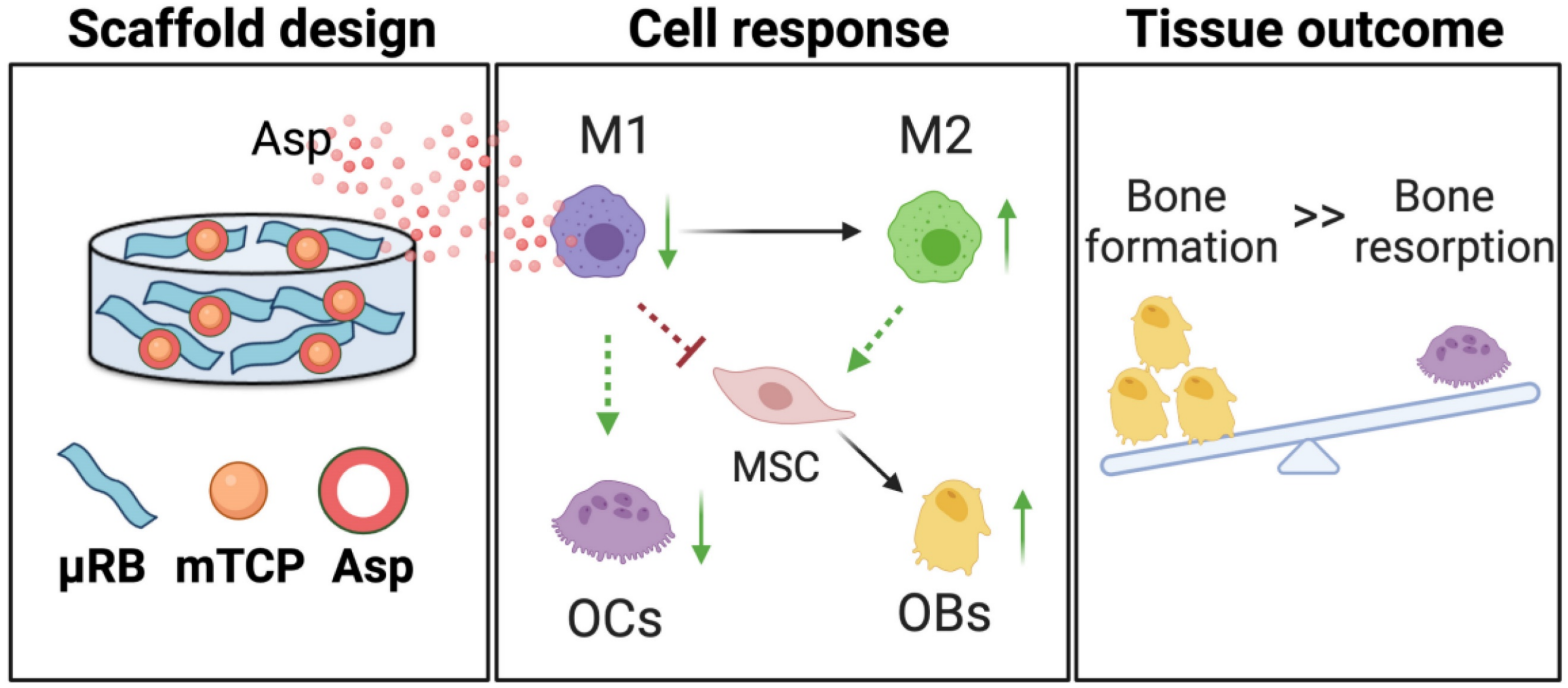
** A schematic summary of how Asp and mTCP in μRB scaffolds synergize to promote desirable cell fates and bone regeneration through immunomodulation.** Schematic created with BioRender.com.

We demonstrate that Asp and mineral particles can synergize to enhance macroporous µRB scaffold-mediated bone regeneration via enhancing Mφ transition from M1 to M2, decreasing OC activation, and promoting MSC osteogenesis (Figure 7). To achieve the maximal benefit, optimizing the dosage of Asp and the type of mineral particle is critical. The findings of this study emphasized the importance of immunomodulation in maximizing the benefit of CaP particles to enhance bone regeneration while avoiding undesirable bone resorption. Given that Asp is an FDA-approved drug with low cost and wide clinical use, it represents an attractive immunomodulator for bone regeneration. This study is novel because it is the first demonstration that the unique combination of µRB scaffolds, mineral particles, and optimized Asp dosage can induce rapid endogenous bone regeneration *in vivo*. One advantage of this approach is that it not only leverages the benefit of each component but also fosters the synergy among the three components. Compared to other regenerative strategies that require the use of exogenous stem cells, this scaffold-only approach is more attractive for clinical translation with reduced cost and ease of fabrication as an off-the-shelf product.

## Supplementary Material

Supplementary figures and tables.
