# Supplementary material for: Aspirin synergizes with mineral particle-coated macroporous scaffolds for bone regeneration through immunomodulation
Source: Theranostics. 2023 Aug 15;13(13):4512–25. doi: 10.7150/thno.85946 (PMC10465219; doi:10.7150/thno.85946)
Supplement: Supplementary file 1 — Supplementary figures and tables. [file thnov13p4512s1.pdf]

Supplemental information

Table S1. Sequences of primers used for qPCR.

Primer Name	Forward Sequence 5' to 3'	Reverse Sequence 5' to 3'
**CD86**	CTGGACTCTACGACTTCACAATG	AGTTGGCGATCACTGACAGTT
**TNF-α**	GACCCTCACACTCAGATCATCTTCT	CCTCCACTTGGTGGTTTGCT
**CD206**	CTCTGTTCAGCTATTGGACGC	CGGAATTTCTGGGATTCAGCTTC
**IL-10**	ATGCTGCCTGCTCTTACTGACTG	CCCAAGTAACCCTTAAAGTCCTGC
**TRAP**	CTGGAGTGCACGATGC CAGCGACA	TCCGTGCTCGGCGATGGACCAGA
**OSCAR**	CTGCTGGTAACGGATCAGCTCCCCAGA	CCAAGGAGCCAGAACCTTCGAAACT
**CTSK**	CT TCCAATACGTGCAGCAGA	TCTTCAGGGCTTTCT CGTTC
**BMP**	GGGACCCGCTGTCTTCTAGT	TCAACTCAAATTCGCTGAGGAC
**ALP**	CCAACTCTTTTGTGCCAGAGA	GGCTACATTGGTGTTGAGCTTTT
**OCN**	CCTGAGTCTGACAAAGCCTTCA	GCCGGAGTCTGTTCACTACCTT
**OPN**	CTTTCACTCCAATCGTCCCTAC	GCTCTCTTTGGAATGCTCAAGT

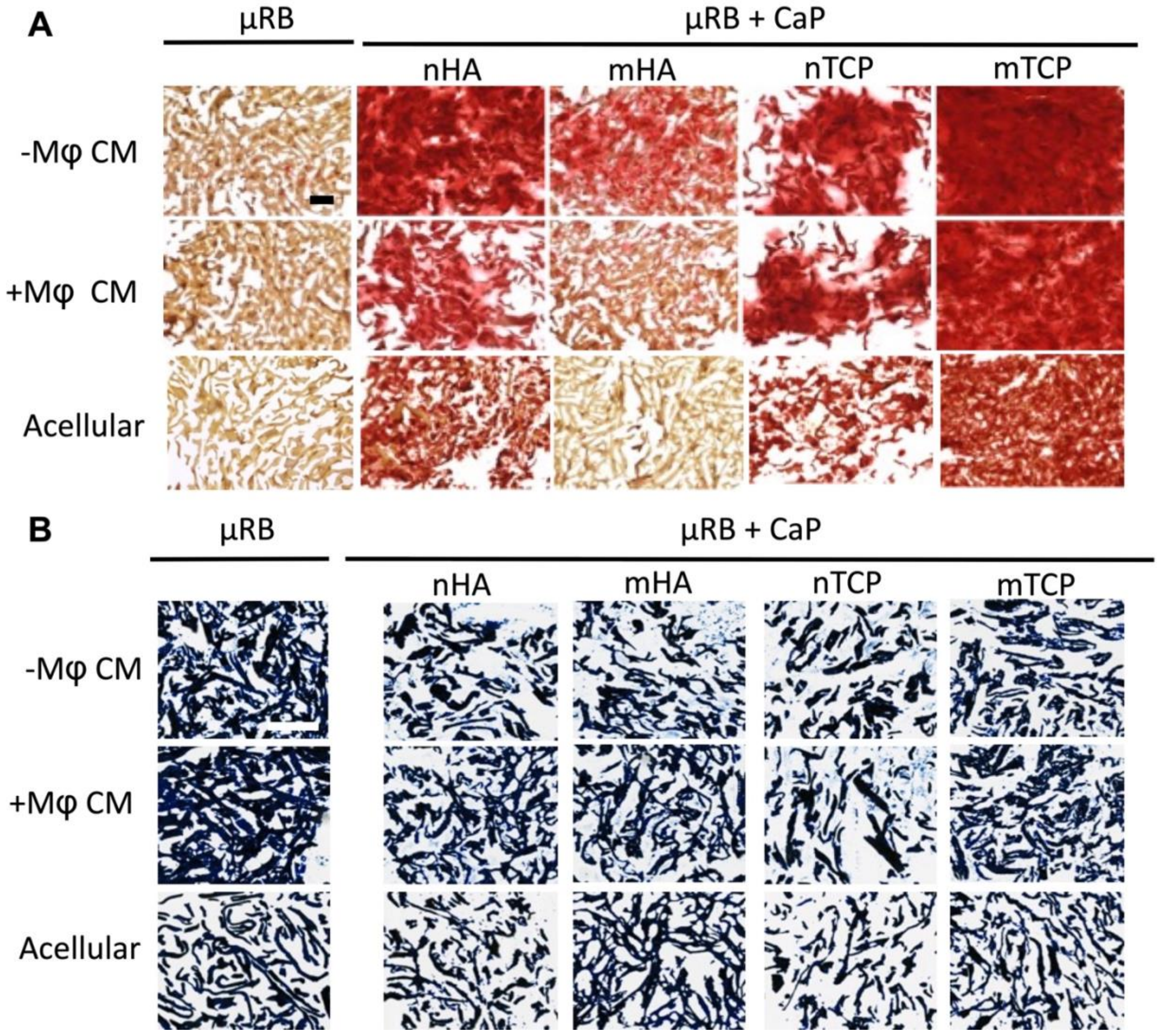

Figure S1: Assessing MSC-based bone formation by staining for mineralization and collagen in μRB scaffolds containing various CaP particles, without or with Mφ conditioned medium (CM). (A) ARS staining of mineralization at day 16. Acellular scaffolds were included as controls to show background staining from various CaP particles. (B) Masson's Trichrome staining for total collagen deposition at day 16. Acellular scaffolds were included as controls to show background staining from μRB scaffolds. Scale bar in (A): 100 μm, (B): 300 μm.

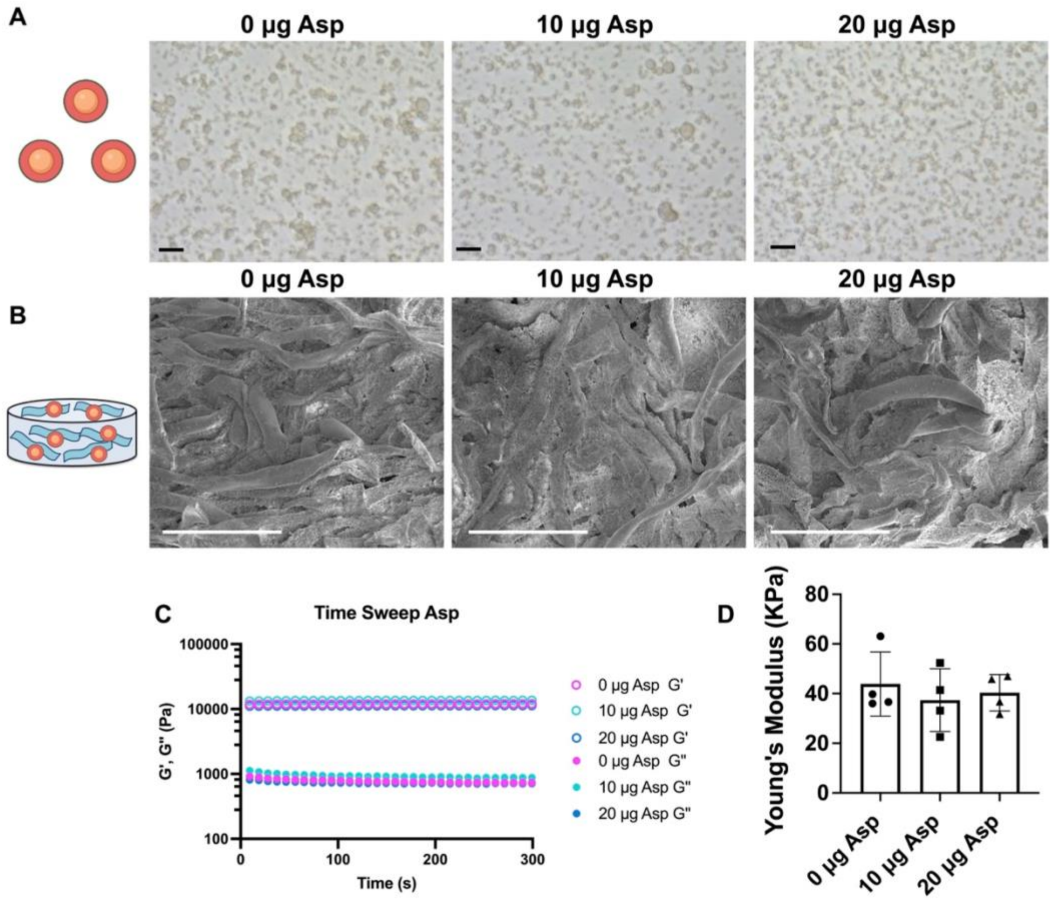

Figure S2: Morphology and rheological properties of Asp-loaded mTCP-μRB scaffold. (A) Morphology of mTCP particles coated without or with varying concentrations of Asp. (B) SEM imaging of mTCP-μRB scaffolds loaded with varying dosages of Asp. (C) Storage modulus (G') and loss modulus (G'') of Asp-loaded CaP-μRB scaffolds. (C) Young's modulus of Asp-loaded CaP-μRB scaffolds. Scale bar in A: 30 μm. Scale bar in B: 500 μm. Data are represented as mean ± S.D. (n = 4/group).

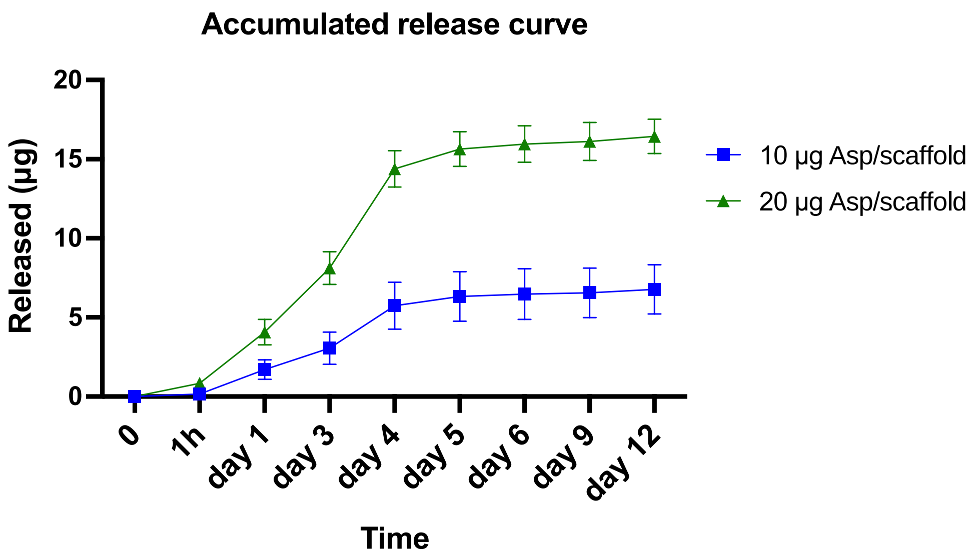

Figure S3: Accumulated release kinetics of Asp from mTCP-μRB scaffold in PBS solution overtime.

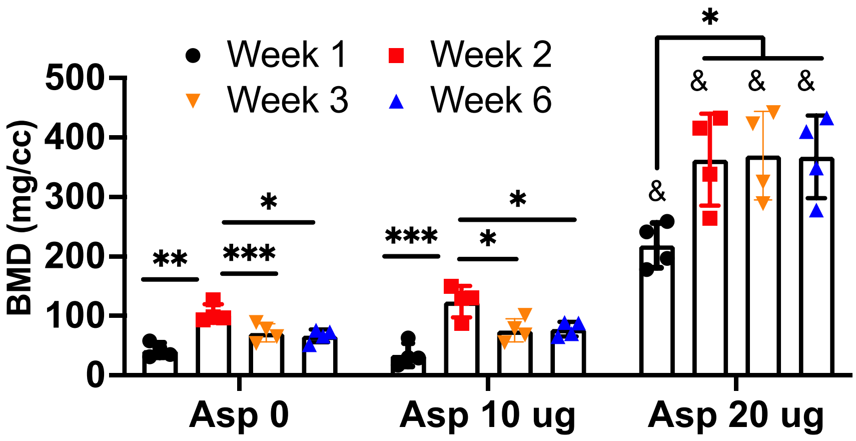

Figure S4: Effects of tuning Asp dosage on modulating bone mineral density (BMD) of regenerated bone in a mouse critical-sized cranial defect model.

* Indicates comparison within the same Asp dosage at varying time points. *: $P<0.05$, **: $P < 0.01$, ***: $P < 0.001$. & indicates statistically significant differences between 20 μg Asp group vs. 0 μg and 10 μg Asp group at the corresponding time points. &: $P < 0.01$.

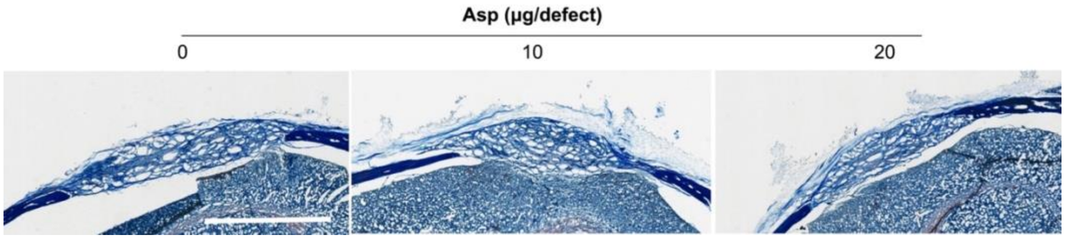

Figure S5: Trichrome staining showing scaffold morphology in the cranial defect at week. The scale bar indicates 2 mm.

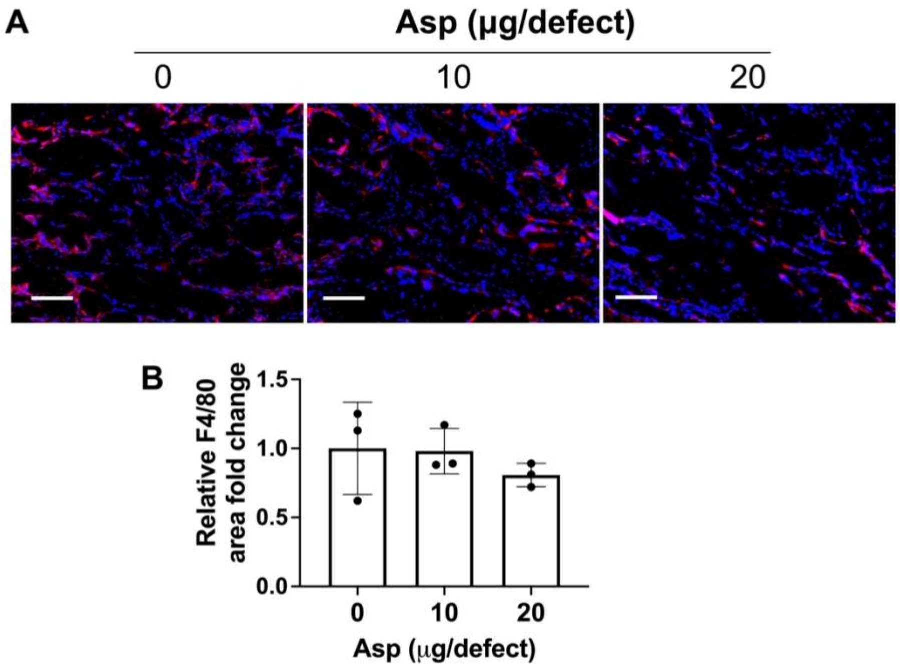

Figure S6: Aspirin does not affect total Mφ infiltration *in vivo* at week 2. (A-B) Representative immunostaining images and quantification of F4/80+ Mφ.
